# Gender differences in tuberculosis patients with comorbidity: A cross-sectional study using national surveillance data and national health insurance claims data in South Korea

**DOI:** 10.1371/journal.pone.0280678

**Published:** 2023-01-20

**Authors:** Daseul Moon, Dawoon Jeong, Young Ae Kang, Hongjo Choi

**Affiliations:** 1 Center for Labor and Health, People’s Health Institute, Seoul, Republic of Korea; 2 Research and Development Center, The Korean Institute of Tuberculosis, Korean National Tuberculosis Association, Cheongju, Republic of Korea; 3 Department of Internal Medicine, Division of Pulmonary and Critical Care Medicine, Severance Hospital, Yonsei University College of Medicine, Seoul, Republic of Korea; 4 Department of Preventive Medicine, Konyang University College of Medicine, Daejeon, Republic of Korea; SMS Medical College and Hospital, INDIA

## Abstract

The coexistence of tuberculosis and other chronic diseases complicates disease management. Particularly, the lack of information on the difference in the prevalence of chronic diseases in tuberculosis based on age and gender can hinder the establishment of appropriate public health strategies. This study aimed to identify age- and gender-based differences in the prevalence of chronic diseases as comorbidities in patients with tuberculosis. An anonymized data source was established by linking the national health insurance claims data to the Korean national tuberculosis surveillance data from 2014 to 2018. The prevalence of chronic diseases was stratified by gender and age (age groups: ≤64, 65–74, and ≥75 years), and the differences in the prevalence of chronic diseases were analyzed by multinomial logistic regression and classified using the Charlson Comorbidity Index. A total of 148,055 patients with tuberculosis (61,199 women and 86,856 men) were included in this study. Among the patients aged ≥65 years, 48.2% were female and 38.1% were male. In this age group, the probability of chronic disease comorbidity was higher in female patients than in male patients. The prevalence of congestive heart failure and dementia as comorbidities in patients with tuberculosis increased more drastically with age in women than in men. Thus, the present study confirmed gender and age differences in the distribution of comorbidities among patients with tuberculosis. A more comprehensive gender-responsive approach for patients with tuberculosis and chronic diseases is required to alleviate the double burden of infectious diseases and non-communicable diseases in an aging society.

## Introduction

Tuberculosis (TB) is an infectious disease that remains the leading cause of death [1], and its control is a major concern worldwide [2,3]. Furthermore, the high prevalence of multimorbidity in patients with TB is one of the main obstacles to its management [4,5]. The coexistence of TB and chronic diseases should be considered a serious public health issue because they can affect the treatment efficacy and health of patients and their subsequent well-being and quality of life in general.

Studies on TB and related comorbidities with non-communicable diseases (NCD) have reported an association between TB and diabetes. For example, the population attributable fraction (PAF) of diabetes for TB onset was approximately 6.2–8.0% in Africa but exceeded 14% in Europe [6]. These values exceeded the PAF levels for human immunodeficiency virus (HIV) infection and malnutrition [6]. TB also has a bidirectional association with chronic obstructive pulmonary disease (COPD) [7]. The risk of developing TB is two to three times higher in patients with COPD [8], and TB patients with COPD have a mortality rate twice as high as that of patients without COPD [9]. In addition, clinical investigations have reported an association between TB and cancer, chronic lung disease, chronic kidney disease or end-stage renal failure, autoimmune hepatitis, gut malabsorption syndromes, acquired immunodeficiency syndrome, gastric bypass or gastrectomy, Crohn’s disease, ulcerative colitis, organ transplants, reticuloendothelial disorders, rheumatoid arthritis, autoimmune disease, psoriasis, alopecia areata, sarcoidosis, and solid organ transplantation [3].

Furthermore, the prevalence of TB with other chronic diseases is significantly affected by socio-economic factors [10–12]. Moreover, TB with other chronic diseases disproportionately affects vulnerable sections of society, including low-income groups, homeless people, and immigrants [13–15]. Therefore, there could be a higher proportion of the double burden of comorbidity of TB and chronic NCDs in such populations, and such a double burden and disease interaction could further exacerbate their vulnerability.

Korea has the fastest growing aging population and the highest incidence of TB among all Organization for Economic Cooperation and Development countries. Hence, understanding multimorbidity among patients with TB is important. Studies conducted in Korea have identified the impact of chronic diseases on TB. The PAF for diabetes was approximately 20%, whereas the PAF for smoking and alcohol consumption was 18.8 and 18.4%, respectively [16]. Another study empirically demonstrated the effect of the coexistence of diabetes and smoking on the increase in the TB-related mortality rate [17]. However, multimorbidity among patients with TB and its distribution in Korea is not well known. The risk and prevalence of chronic diseases is higher in women than in men, and among the older adult population in Korea [18]. Additionally, in the older population in Korea, the number of healthy life years of the average life expectancy is longer for men (81.4%) than for women (77.7%), as of 2020 [19]. Hence, gender and age could play a significant role in the prevalence of chronic disease comorbidities among patients with TB in Korea.

Although NCD multimorbidity among patients with TB is well known, relevant empirical studies have been conducted only in low- and middle-income countries with a high incidence of TB and mortality rates [5,20–23]. As part of such efforts, the present study examined comorbidities at the time of TB onset in individuals in Korea. Moreover, the age- and gender-based differences in the prevalence of chronic diseases in patients with TB have not been characterized earlier. Therefore, this study evaluated age- and gender-based differences in the prevalence of chronic disease comorbidities among drug-susceptible TB patients with TB. This study found that the prevalence of comorbidities among female patients with TB increases with age but not among male patients.

## Materials and methods

### Study design and measurements

This was a cross-sectional study of patients with TB in Korea between 2014 and 2018. The anonymized data source was established by linking TB surveillance data to national health insurance claims data using personal identifiable information. Patients with TB registered in the surveillance system were included in this study. Among the 151,112 patients registered in the TB surveillance system, 2,886 were excluded due to missing information on the type of health institution (n = 4), treatment results (n = 455), income (n = 2,285), region (n = 142), and unrealistic treatment periods (n = 171). Finally, 148,055 patients (61,199 women and 86,856 men) registered in the TB surveillance system were included in the study.

This study was conducted in accordance with the 2008 Declaration of Helsinki and approved by the independent Institutional Review Board of Yonsei University Health System (IRB number: 4-2019-0917). Written informed consent was not obtained because the patients’ records and information were anonymized and de-identified prior to the analysis. The need for informed consent was waived by the institutional review board of the Yonsei University Health System.

The major independent variable was gender. The main stratified variable was age, which was divided into three groups: ≤64, 65–74, and ≥75 years. The Charlson Comorbidity Index (CCI), a typical chronic disease index, was used as an outcome variable [24]. Because the CCI values typically show a left-skewed distribution with most values close to 0, the values were measured and classified into groups of 0, 1, 2, and ≥3 points. To test the differences in the distribution of each chronic disease, each individual disease group included in the calculation of CCI was considered an outcome variable. Covariates included region of residence (metropolitan and others), nationality (Korean and others), household income level (0 = medical aid beneficiary, 1–5 = health insurance beneficiary; with 0 being the lowest income level, and 5 being the highest income level), lesions of TB (pulmonary and extrapulmonary TB), previous TB history (new and previously treated TB cases), notified health institution (health center, hospital/clinic, and both), results of acid-fast bacilli (AFB) smear microscopy (positive, negative, and unknown), results of AFB culture (positive, negative, and unknown), and disability (non-disabled and disabled).

### Statistical analysis

The baseline characteristics of the patients were stratified by gender and age (age groups: ≤ 64, 65–74, and ≥ 75 years) and expressed as differences in the distribution of each covariate. Differences in distribution were estimated using Pearson’s chi-square test or the Conchran-Armitage test. The prevalence of individual diseases included in the CCI was divided by gender and age, and differences in distribution were analyzed using Pearson’s chi-square test. To analyze the distribution of CCI scores stratified by gender and age, a multinomial logistic regression model was used.

The covariate region of residence, nationality, household income level, disability, TB lesions, previous TB history, notified health institution, AFB smear results, and AFB culture results were included in the model and adjusted accordingly. Point estimates were calculated as relative risk ratios. Age-stratified multivariate logistic regression analysis was performed for gender-based differences in the prevalence of each disease group. The probability of having a particular disease in each age group among men relative to women was presented as odds ratios (OR) and 95% confidence intervals (CI). All statistical analyses were performed with STATA/MP4 version 17 (StataCorp LLC, College Station, TX, USA) with *P*-value < 0.05 as the criterion of statistical significance.

## Results

The percentage of women in the ≤64 and 65–74 years age groups decreased from 57.2% in 2014 to 45.7% in 2018 and from 14.3% in 2014 to 13.3% in 2018, respectively (*p* < 0.001), whereas the percentage of women in the ≥75 years age group continued to increase from 28.6% in 2014 to 41.1% in 2018 (*p* < 0.001). Regarding the distribution by age group, a lower percentage of both men and women in all age groups lived in metropolitan cities (Table 1). Regarding household income level, the percentage of those belonging to the lowest-income (0) and high-income (5) groups tended to increase in the older population compared with those aged ≤64 years (*p* < 0.001). The results showed that the distributions of all covariates in all age groups were significantly different between men and women (Table 2). However, the distribution of the prevalence of major comorbidities differed between genders. In all age groups, comorbidities that were more prevalent among men than those among women included mild liver disease, hemiplegia or paraplegia, cancer, moderate or severe liver disease (no difference in the distribution of prevalence among those aged ≥75 years), and metastatic solid tumors (no difference in the distribution of prevalence among those aged ≤64 years). Compared to that among male patients, the only comorbidity that was more prevalent among women in all age groups was rheumatologic disease. The prevalence of some diseases, including congestive heart failure and dementia, increased with age to a greater extent in women than in men. Moreover, there were no gender-based differences in the ≤64 and ≥75 years age groups for diabetes with chronic complications; however, the prevalence was higher among women than that among men in the 65–74 years age group. In contrast, the prevalence of COPD was higher among women than that among men in the ≤64 and 65–74 years age groups but higher among men in the ≥75 years age group. With respect to the CCI score, which measured the sum of all comorbidities, there were more men with comorbidities in the ≤64 years age group and more women with comorbidities in the 65–74 and ≥75 years age groups with a score of ≥2 points (Table 3).

**Table 1:** All registered patients with tuberculosis by year, age group, and gender.

Notified year	Female						Male					
	≤64		65–74		≥75		≤64		65–74		≥75	
	n	%	n	%	n	%	n	%	n	%	n	%
2014	8,089	57.2	2,018	14.3	4,041	28.6	13,013	66.2	3,142	16.0	3,501	17.8
2015	6,815	53.7	1,823	14.4	4,043	31.9	11,712	64.5	2,959	16.3	3,492	19.2
2016	6,212	50.7	1,686	13.8	4,353	35.5	10,897	62.0	2,971	16.9	3,709	21.1
2017	5,716	49.8	1,556	13.6	4,200	36.6	9,572	59.3	2,657	16.5	3,901	24.2
2018	4,862	45.7	1,413	13.3	4,372	41.1	8,583	56.0	2,611	17.0	4,136	27.0

**Table 2:** Baseline characteristics of female and male patients with tuberculosis by age group.

Variables		≤64					65–74					≥75				
		Female		Male			Female		Male			Female		Male		
		n	%	n	%	*p*-value	n	%	n	%	*p*-value	n	%	n	%	*p*-value
Region	Metropolitan	15,232	48.06	24,711	45.95	<0.001	3,512	41.34	6,237	43.49	0.001	6,805	32.39	6,737	35.95	<0.001
	Others	16,462	51.94	29,066	54.05		4,984	58.66	8,103	56.51		14,204	67.61	12,002	64.05	
Nationality	Korean	29,989	94.62	51,263	95.33	<0.001	8,372	98.54	14,191	98.96	0.005	20,888	99.42	18,691	99.74	<0.001
	Others	1,705	5.38	2,514	4.67		124	1.46	149	1.04		121	0.58	48	0.26	
Household income level (0 = lowest)	0	1,468	4.63	3,947	7	<0.001	760	8.95	1,327	9.25	<0.001	2,629	12.51	1,675	8.94	<0.001
	1	5,794	18.28	9,654	17.95		1,238	14.57	2,255	15.73		3,236	15.4	2,016	10.76	
	2	6,094	19.23	10,476	19.48		831	9.78	1,991	13.88		2,066	9.83	1,594	8.51	
	3	6,365	20.08	10,925	20.32		1,215	14.3	2,127	14.83		2,527	12.03	2,260	12.06	
	4	6,125	19.33	9,805	18.23		1,766	20.79	3,107	21.67		3,359	15.99	3,421	18.26	
	5	5,848	18.45	8,970	16.68		2,686	31.61	3,533	24.64		7,192	34.23	7,773	41.48	
Lesions of TB	PTB	22,395	70.66	44,461	82.68	<0.001	6,195	72.92	11,909	83.05	<0.001	16,755	79.75	15,144	80.82	0.008
	EPTB	9,299	29.34	9,316	17.32		2,301	27.08	2,431	16.95		4,254	20.25	3,595	19.18	
Type of TB	New case	28,800	90.87	47,110	87.6	<0.001	7,761	91.35	11,794	82.25	<0.001	19,349	92.1	15,677	83.66	<0.001
	Previously treated case	2,894	9.13	6,667	12.4		735	8.65	2,546	17.75		1,660	7.9	3,062	16.34	
Notified health institutions	Health center	2,101	6.63	4,546	8.45	<0.001	355	4.18	733	5.11	0.006	565	2.69	679	3.62	<0.001
	Hospital/clinic	29,570	93.3	49,179	91.45		8,135	95.75	13,595	94.8		20,418	97.19	18,044	96.29	
	Both	23	0.07	52	0.1		6	0.07	12	0.08		26	0.12	16	0.09	
AFB smear	positive	17,589	55.5	31,722	58.99	<0.001	4,537	53.4	8,664	60.42	<0.001	10,750	51.17	10,878	58.05	<0.001
	negative	6,190	19.53	14,848	27.61		2,220	26.13	4,048	28.23		7,194	34.24	5,829	31.11	
	unknown	7,915	24.97	7,207	13.4		1,739	20.47	1,628	11.35		3,065	14.59	2,032	10.84	
AFB culture	positive	12,141	38.31	26,117	48.57	<0.001	3,865	45.49	7,250	50.56	<0.001	11,265	53.62	9,848	52.55	<0.001
	negative	10,556	33.31	18,090	33.64		2,500	29.43	4,719	32.91		5,264	25.06	5,573	29.74	
	unknown	8,997	28.39	9,570	17.8		2,131	25.08	2,371	16.53		4,480	21.32	3,318	17.71	
Disability	non-disabled	29,934	94.45	48,261	89.74	<0.001	6,885	81.04	11,072	77.21	<0.001	16,971	80.78	14,217	75.87	<0.001
	disabled	1,760	5.55	5,516	10.26		1,611	18.96	3,268	22.79		4,038	19.22	4,522	24.13	

* TB = tuberculosis, PTB = pulmonary tuberculosis, EPTB = extrapulmonary tuberculosis, AFB = acid fast bacilli.

**Table 3:** Prevalence of various comorbidities in female and male tuberculosis patients by age group.

		≤64					65–74					≥75					
		Female	Male			Female	Male			Female	Male						
		n	%	n	%	*p*-value	n	%	n	%	*p*-value	n	%	n	%	*p*-value
Congestive heart failure		778	2.45	1,850	3.44	<0.001	1,137	13.38	1,802	12.57	0.075	5,190	24.7	3,927	20.96	<0.001
Dementia		211	0.67	473	0.88	0.001	667	7.85	879	6.13	<0.001	3,905	18.59	2,647	14.13	<0.001
Chronic pulmonary disease		14,915	47.06	23,186	43.12	<0.001	5,650	66.5	9,351	65.21	0.047	14,746	70.19	13,487	71.97	<0.001
Rheumatologic diseases		327	1.03	217	0.4	<0.001	215	2.53	176	1.23	<0.001	269	1.28	166	0.89	<0.001
Mild liver disease		1,000	3.16	2,591	4.82	<0.001	424	4.99	767	5.35	0.239	653	3.11	676	3.61	0.006
Diabetes with chronic complications		46	0.15	90	0.17	0.431	33	0.39	27	0.19	0.004	33	0.16	35	0.19	0.474
Hemiplegia or paraplegia		106	0.33	402	0.75	<0.001	181	2.13	367	2.56	0.041	605	2.88	612	3.27	0.026
Renal disease		148	0.47	304	0.57	0.056	86	1.01	154	1.07	0.659	148	0.7	180	0.96	0.005
Cancer		643	2.03	1,275	2.37	0.001	291	3.43	1,063	7.41	<0.001	252	1.2	756	4.03	<0.001
Moderate or severe liver disease		23	0.07	173	0.32	<0.001	13	0.15	54	0.38	0.003	27	0.13	31	0.17	0.336
Metastatic solid tumor		181	0.57	282	0.52	0.369	92	1.08	223	1.56	0.003	92	0.44	177	0.94	<0.001
PLHIV		16	0.05	238	0.44	<0.001	5	0.06	17	0.12	0.160	1	0	14	0.07	<0.001
CCI score	0	15,710	49.57	27,882	51.85	<0.001	2,128	25.05	3,889	27.12	<0.001	3,806	18.12	3,505	18.7	<0.001
	1	13,593	42.89	20,287	37.72		4,071	47.92	6,812	47.5		8,378	39.88	8,341	44.51	
	2	944	2.98	2,180	4.05		634	7.46	922	6.43		2,036	9.69	1,471	7.85	
	3 or above	1,447	4.57	3,428	6.37		1,663	19.57	2,717	18.95		6,789	32.31	5,422	28.93	

* PLHIV = people living with HIV/AIDS, CCI = Charlson comorbidity index.

**Fig 1:**
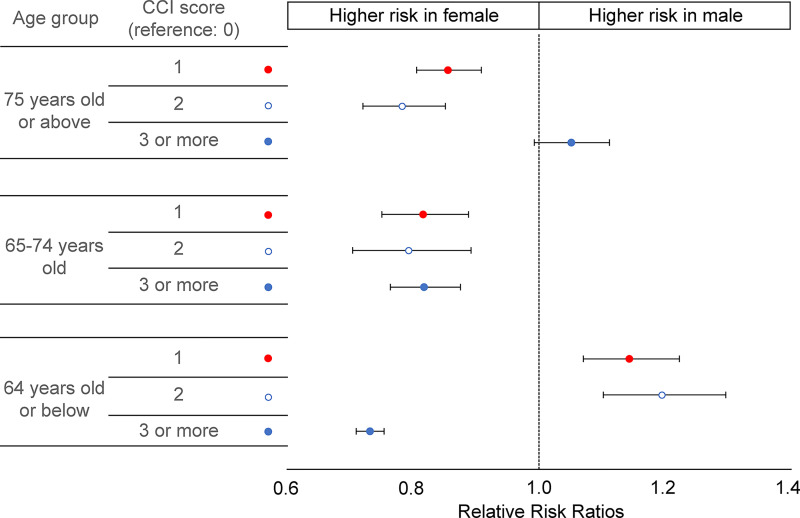
Associations between the Charlson comorbidity index and gender by age group. Multinomial logistic regression model (reference: Gender = female, CCI = 0; adjusted for region, nationality, household income, lesions of tuberculosis, type of tuberculosis, notified health institution, and acid-fast bacilli smear and culture results). CCI = Charlson comorbidity index.

**Fig 2:**
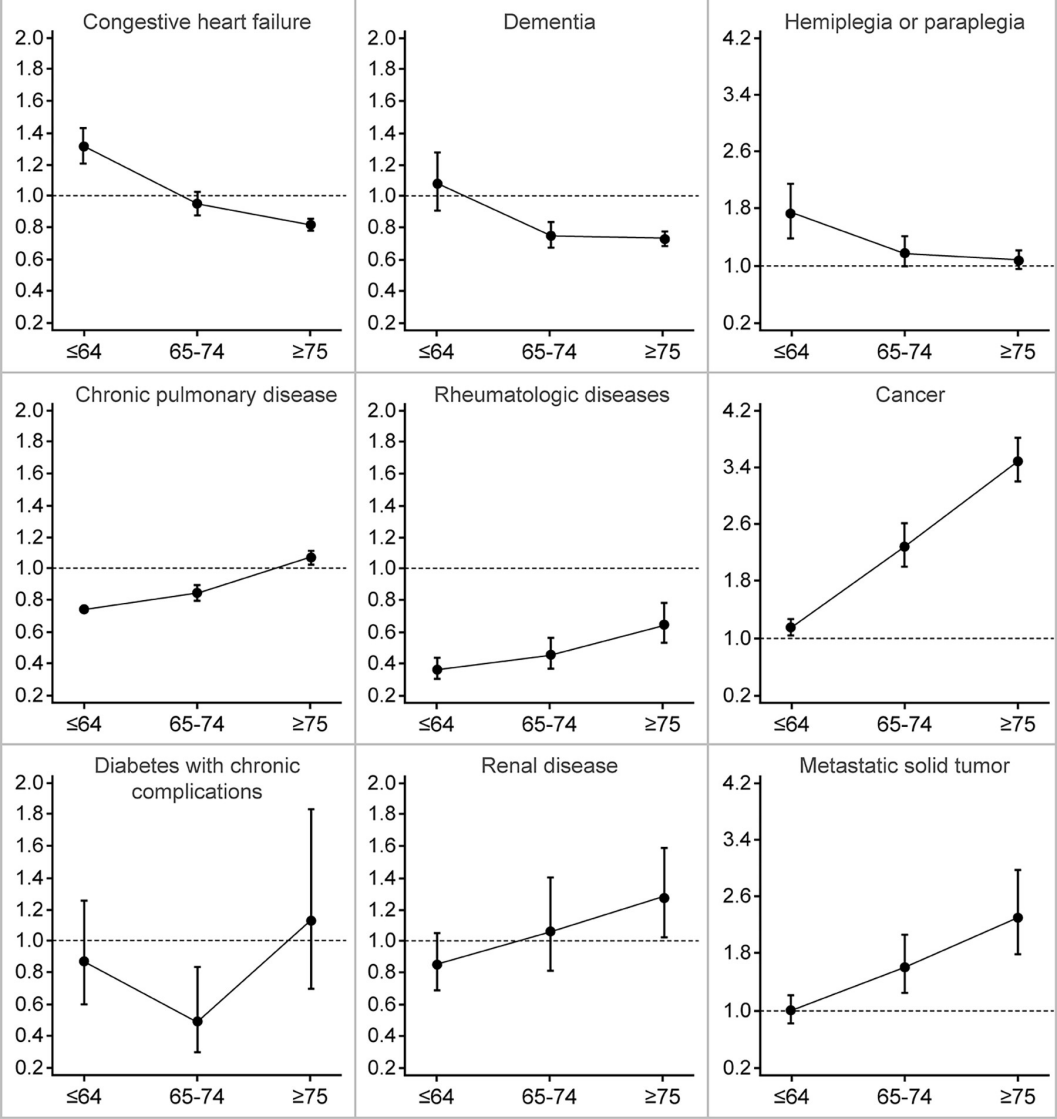
Association between comorbidities and gender by age group. Logistic regression model (reference: Female; adjusted for region, nationality, household income, lesions of tuberculosis, type of tuberculosis, notified health institution, and acid-fast bacilli smear and culture results; y axis = adjusted odds ratios; x axis = age group; vertical solid line = 95% confidence intervals).

In the multinomial logistic regression model analysis, men had a higher probability of CCI scores of 1 or 2 than women, but women had a higher probability of CCI score of ≥3 than men, with a CCI score of 0 as a reference, in the ≤64 years age group (RRR = 0.73, 95% CI = 0.71–0.75). In the ≥75 years age group, there was no difference in the probability of CCI scores of ≥3 between men and women. In the 65–74 and ≥75 years age groups, women had a higher probability of CCI scores of 1, 2, or ≥3 than men, using CCI score of 0 as reference (Fig 1 and S1 Table). Investigation of age- and gender-based differences in the distribution of individual diseases using multivariate logistic regression analysis revealed an increased risk of congestive heart failure and dementia with increasing age among women. In all age groups, the risk of rheumatologic disease was also higher among women than that among men. The risk of cancer and metastatic solid tumors was higher among men than that among women in all age groups, except for the ≤64 years age group, which was not statistically significant (Fig 2, S2 Table).

## Discussion

Owing to the increasing global aging population, studying the effect of comorbidities on the management of chronic infectious diseases, such as TB, has become vital. Identifying gender-based differences in the prevalence of TB and comorbidities can provide a framework for future disease management strategies. The present study used the distribution of CCI scores and revealed an increasing prevalence of comorbidities among female patients with TB with increasing age. These findings could be due to the differences between genders in the prevalence of specific diseases, including congestive heart failure, dementia, and rheumatologic diseases, with increasing age.

In the present study, we observed a higher prevalence of multimorbidity among older female patients with TB. However, previous studies reported the opposite, with a greater focus on multimorbidity among male patients, and those that analyzed gender-based differences in multimorbidity among patients with TB are limited. A Taiwanese study on the gender-based differences in the risk of dementia among TB patients reported that the risk of dementia stratified by TB infection was not significantly different between genders, except among patients aged 50–64 years, in which male patients with TB had a higher risk of dementia [25]. However, as these studies used different statistical analysis models, their findings should be compared cautiously. For example, the major independent variable in the present study was gender, whereas that in the Taiwanese study was TB; the Taiwanese study performed a gender-stratified analysis. The conceptualization and operationalization of the dependent variables also varied: the CCI score and chronic disease at the time of TB onset (i.e., multimorbidity) were the major dependent variables in the present study, whereas the presence of dementia (i.e., comorbidity) was used as the dependent variable in the Taiwanese study. Furthermore, only patients with drug-susceptible TB were included as the target population in the present study, whereas all newly diagnosed TB patients were included in the Taiwanese study. A South African study on NCD multimorbidity among patients with TB in public primary care clinics also reported that the risk was higher among males [22]; however, direct comparison with the findings in the present study is difficult because the study did not stratify patients by age.

However, considering the differences in multimorbidity between older Korean male and female patients, the results of the present study are plausible. A previous study that analyzed the distribution of chronic disease multimorbidity among the older population in Korea [18] reported that the majority of patients with at least three chronic diseases were women in the 65–80 years age group; however, the number of these patients of both genders was similar in ≥78 years age group, with a higher number of male patients in ≥80 years age group. Jeong et al. also observed that the prevalence of heart failure, rheumatologic diseases, and dementia was higher among women than that among men, whereas the prevalence of cancer was higher among men than that among women. This distribution supports the findings of the present study, showing that the risk of congestive heart failure, dementia, and rheumatologic diseases was higher among older women, and the risk of cancer and metastatic solid tumors was higher among older men. However, this study used data from 2011 and did not include TB in its analysis.

Another possible explanation for the relatively higher CCI scores among older women than those among older men may be the harvesting effect. In other words, older male TB patients with chronic disease may have a relatively higher severity of disease [26] than older women, and as a result, excess deaths may have occurred among males. However, caution should be exercised when interpreting these differences because of their biological and epidemiological characteristics. Differences in the severity, survival, and multimorbidity of elderly male and female patients with TB could be attributed to differences in their health behaviors. An investigation of the differences in healthcare utilization among the older Korean population showed that older women had higher healthcare utilization than older men, with especially high utilization of inpatient and outpatient care [27–29]. However, another study [27] reported that their total medical expenditure was relatively lower, which could be related to the relatively low socioeconomic status of older women. In Korea, older women have the lowest income [30]. This indicates that older women utilize healthcare services with relatively lower quality, which might have simultaneously caused multiple diseases and resulted in a low quality of life.

The implications of this study are as follows: First, a comprehensive approach for TB and chronic diseases was required for older patients with TB in Korea, rather than just disease-specific strategies [7,31]. The epidemiological transition has already suggested a new approach to health [3], which is not different from TB. In particular, considering the rapid increase in the aging population and high incidence of TB in Korea, the present study suggests a need for TB management in Korea to respond quickly to such a transition. Second, the present study was the first to investigate gender-based differences in the risk of chronic disease multimorbidity among older patients with TB in Korea. The findings confirmed that among older patients with TB, the risk of chronic disease comorbidity was higher in female patients than that in male patients. These findings demonstrate the limitations of male-centric interventions and conventional treatments, which assume that the prevalence of TB is higher in male patients.

The present study has some limitations. First, as mentioned earlier, the higher CCI scores among older female patients with TB could be due to the harvesting effect in older male patients. However, considering that the risk of multimorbidity faced by male and female TB patients may vary depending on the disease severity and that the prevalence of cancer and metastatic solid tumors was high among men (Fig 2), the gender-based difference in multimorbidity should be considered important, even with the harvesting effect. Second, data on sex and gender were used interchangeably based on the availability of data. Although many studies have already indicated such limitations [32], the present study was unable to overcome this problem owing to limitations in the data used. Despite this, interpreting the findings in the study as gender-based differences is still valid since the findings cannot be explained solely by the biological differences between genders. Third, the study missed TB progression [33] and delayed diagnosis and treatment of TB [10] which are associated with prevalence of comorbidities among the study population in statistical models due to data limitation. Nevertheless, this study attempted to measure the past prevalence of TB (i.e., new cases and previously treated cases) of patients at the time of cohort registration and smear microscopy as proxy variables reflect TB progression and delayed diagnosis, respectively. Fourth, the study did not include other mediating factors that may cause gender-based differences, such as healthcare utilization by older patients with TB. Consequently, the mechanism proposed in this study remains a hypothesis. Future studies should consider these factors. Finally, the generalization of the findings from the present study should be done cautiously since all participants in the present study were from Korea.

## Conclusions

The double burden of infectious disease and NCD poses a significant challenge to healthcare response approaches. The limitations of traditional disease-specific vertical approaches in healthcare service delivery for prevention, treatment, and recovery from specific infectious diseases, as well as the acute approach to disease classification, have become obvious. Therefore, considering TB and chronic disease multimorbidity and the bidirectional association between the two conditions, there is a need for an integrative approach to TB and chronic diseases to develop TB management strategies. Strategies that do not consider such a double burden and focus solely on a single disease cannot enhance the well-being of the older population, which can cause unexpected complications and increase mortality in the older population. Second, and more importantly, the gender-transformative approach, beyond the gender-responsive approach, is essential for developing disease management strategies, from data collection to the derivation of evidence. The findings in the present study showed that not all TB patients have the same chronic disease as comorbidity and that there are gender-based differences in the distribution of such comorbidities. These findings acknowledge the needs of each gender for the management of health in patients with TB. Only when both approaches, a comprehensive approach for TB and chronic disease and a gender-responsive approach, are included in the development of TB management strategies can the double burden of infectious disease and NCD be alleviated in an aging society, which can ultimately improve the quality of life of the older population.

## Supporting information

S1 Table Association between Carlson comorbidity index and gender by age group. (DOCX)

S2 Table Associations between comorbidities and gender by age group. (DOCX)
