# Supplementary material for: Gender differences in tuberculosis patients with comorbidity: A cross-sectional study using national surveillance data and national health insurance claims data in South Korea
Source: PLoS One. 2023 Jan 20;18(1):e0280678. doi: 10.1371/journal.pone.0280678 (PMC9858888; doi:10.1371/journal.pone.0280678)
Supplement: S1 Table — (DOCX) [file pone.0280678.s001.docx]

**Supporting information**

S1 Table. Association between Carlson comorbidity index and gender by age group.

| Age group | CCI score (reference: 0) | RRR (reference: female) | 95% CI | | |
| --- | --- | --- | --- | --- | --- |
| ≤64 | 0 | 1.000 |  |  |  |
|  | 1 | 1.144 | 1.070 | ~ | 1.223 |
|  | 2 | 1.195 | 1.102 | ~ | 1.296 |
|  | ≥3 | 0.732 | 0.710 | ~ | 0.754 |
| 65-74 | 0 | 1.000 |  |  |  |
|  | 1 | 0.816 | 0.751 | ~ | 0.888 |
|  | 2 | 0.793 | 0.705 | ~ | 0.892 |
|  | ≥3 | 0.817 | 0.764 | ~ | 0.875 |
| ≥75 | 0 | 1.000 |  |  |  |
|  | 1 | 0.855 | 0.806 | ~ | 0.908 |
|  | 2 | 0.783 | 0.720 | ~ | 0.851 |
|  | ≥3 | 1.051 | 0.993 | ~ | 1.112 |

Multinomial logistic regression model (reference: gender=female, CCI=0; adjusted for region, nationality, household income, lesions of tuberculosis, type of tuberculosis, notified health institution, and acid-fast bacilli smear and culture results). CCI=Charlson comorbidity index. RRR=relative risk ratio. CI=confidential interval.
