# Supplementary material for: Gender differences in tuberculosis patients with comorbidity: A cross-sectional study using national surveillance data and national health insurance claims data in South Korea
Source: PLoS One. 2023 Jan 20;18(1):e0280678. doi: 10.1371/journal.pone.0280678 (PMC9858888; doi:10.1371/journal.pone.0280678)
Supplement: S2 Table — (DOCX) [file pone.0280678.s002.docx]

**Supporting information**

S2 Table. Associations between comorbidities and gender by age group.

|  | ≤64 | | | | | 65-74 | | | | | ≥75 | | | | |
| --- | --- | --- | --- | --- | --- | --- | --- | --- | --- | --- | --- | --- | --- | --- | --- |
|  | coefficient | 95% CI | | | p-value | coefficient | 95% CI | | | p-value | coefficient | 95% CI | | | p-value |
| Congestive heart failure | 1.312 | 1.202 | ~ | 1.433 | <0.001 | 0.951 | 0.876 | ~ | 1.032 | 0.230 | 0.815 | 0.777 | ~ | 0.855 | <0.001 |
| Dementia | 1.074 | 0.907 | ~ | 1.272 | 0.405 | 0.749 | 0.672 | ~ | 0.834 | <0.001 | 0.732 | 0.693 | ~ | 0.773 | <0.001 |
| Hemiplegia or paraplegia | 1.729 | 1.386 | ~ | 2.156 | <0.001 | 1.179 | 0.979 | ~ | 1.421 | 0.082 | 1.075 | 0.956 | ~ | 1.208 | 0.226 |
| Chronic pulmonary disease | 0.740 | 0.719 | ~ | 0.762 | <0.001 | 0.843 | 0.795 | ~ | 0.894 | <0.001 | 1.068 | 1.021 | ~ | 1.117 | 0.004 |
| Rheumatologic diseases | 0.361 | 0.303 | ~ | 0.430 | <0.001 | 0.453 | 0.368 | ~ | 0.556 | <0.001 | 0.644 | 0.528 | ~ | 0.785 | <0.001 |
| Cancer | 1.148 | 1.041 | ~ | 1.265 | 0.006 | 2.282 | 1.994 | ~ | 2.612 | <0.001 | 3.483 | 3.009 | ~ | 4.031 | <0.001 |
| Diabetes with chronic complications | 0.869 | 0.602 | ~ | 1.255 | 0.455 | 0.494 | 0.292 | ~ | 0.835 | 0.008 | 1.128 | 0.695 | ~ | 1.831 | 0.626 |
| Renal disease | 0.848 | 0.688 | ~ | 1.046 | 0.123 | 1.060 | 0.804 | ~ | 1.397 | 0.680 | 1.273 | 1.019 | ~ | 1.590 | 0.034 |
| Metastatic solid tumor | 1.002 | 0.827 | ~ | 1.214 | 0.984 | 1.604 | 1.249 | ~ | 2.059 | <0.001 | 2.301 | 1.781 | ~ | 2.974 | <0.001 |

Logistic regression model (reference: female; adjusted for region, nationality, household income, lesions of tuberculosis, type of tuberculosis, notified health institution, and acid-fast bacilli smear and culture results). CI=confidential interval.
